# Combined probiotics attenuate chronic unpredictable mild stress-induced depressive-like and anxiety-like behaviors in rats

**DOI:** 10.3389/fpsyt.2022.990465

**Published:** 2022-09-07

**Authors:** Li Huang, Xia Lv, Xiaolei Ze, Zewei Ma, Xuguang Zhang, Ruikun He, Junting Fan, Meilin Zhang, Boran Sun, Fang Wang, Huan Liu

**Affiliations:** ^1^Department of Nutrition and Food Science, School of Public Health, Tianjin Medical University, Tianjin, China; ^2^Tianjin Key Laboratory of Environment, Nutrition, and Public Health, Center for International Collaborative Research on Environment, Nutrition, and Public Health, Tianjin Medical University, Tianjin, China; ^3^BYHEALTH Institute of Nutrition & Health, Guangzhou, China; ^4^Department of Epidemiology and Statistics, School of Public Health, Tianjin Medical University, Tianjin, China; ^5^Department of Pathogen Biology, School of Basic Medical Sciences, Tianjin Medical University, Tianjin, China

**Keywords:** chronic unpredictable mild stress, *Lactobacillus rhamnosus* HN001, *Bifidobacterium animalis* subsp. *lactis* HN019, neurotransmitters, inflammatory factors, gut microbiota

## Abstract

Increasing evidence indicated that probiotics can be effective in improving behaviors similar to depression and anxiety disorders. However, the underlying mechanisms remain unclear, as is the effects of single vs. combined probiotics on depression and anxiety. This study aimed to determine whether combined probiotics could attenuate depressive-like and anxiety-like behavior induced by chronic unpredictable mild stress (CUMS) and its potential mechanisms. Rats underwent CUMS treatment and then administered *Lactobacillus rhamnosus* HN001 (HN001) or *Bifidobacterium animalis* subsp. *lactis* HN019 (HN019), alone or in combination. Levels of neurotransmitters, inflammatory factors, and the gut microbiota were measured. HN001 and (or) HN019 treatment improved depressive-like and anxiety-like behavior in rats, including increased moving distance and exploratory behavior (*p* < 0.05). In addition, altered gut microbiota structure induced by CUMS was amended by HN001 and/or HN019 (*p* < 0.05). HN001 and/or HN019 intervention also remarkably normalized levels of 5-HT, DA, NE, HVA, DOPAC, HIAA, TNF-α, IL-6, IL-18 and IL-1β in CUMS rats (*p* < 0.05). Furthermore, the effects of combined probiotics on decreasing inflammation and improved gut microbiota (Chao1 index and ACE index, *p* < 0.05) were superior to the single probiotics. Moreover, spearman analysis showed a certain correlation between the different microbiota, such as Firmicutes, Bacteroidetes, Verrucomicrobias, Proteobacterias and Actinobacterias, and inflammation and neurotransmitters. These findings suggested that CUMS induced depressive and anxiety-like behaviors can be alleviated by the combination of probiotics, which was possibly associated with the alterations in the gut microbiota composition and increased neurotransmitters and decreased inflammatory factors.

## Introduction

Depression is a predominant and recurrent mental illness that is characterized by anhedonia, depressed mood, and markedly increased suicide rates (1). According to the World Health Organization, presently more than 300 million people being depression and the incidence is still growing (2). As a major contributor to healthcare costs, depression will become the world's leading cause of disease burden by the year 2030 (3). However, the exact etiology and pathogenesis of depression is complex and difficult to determine. After decades of extensive research, several hypotheses regarding the pathogenesis of depression have been proposed, such as the neurotransmitter hypothesis, neuronal circuit hypothesis, and the proinflammatory cytokine hypothesis (4). Currently, commonly antidepressants have large adverse effects and are ineffective for 30–40% depression patients (5, 6). Therefore, there remains an urgent need to find effective and safe antidepressant interventions for treating depression. Recently, studies found that a significant association between the gut microbiota and depression (7, 8). Zheng et al. (9), reported that the gut microbiota might influence the development of depressive-like behaviors by regulating glycerophospholipid metabolism in the gut-brain axis. Other studies showed altered gut microbiota composition and reduced abundance and diversity in depression patients compared with healthy individuals (10, 11), a finding supported by animal studies (12). Moreover, fecal transplantation from depressed patients or mice into germ-free mice replicated depressive symptoms (13). Also, the use of probiotics to modulate the gut microbiota could significantly relieve depression-like symptoms (14, 15). Together, these studies indicated that changes in the gut microbiota may be contribute to the pathogenesis of depression. As live microorganisms from food, probiotics can benefit host health by improving the balance of intestinal flora after sufficient ingestion (16). It appears that at least some probiotics can support brain function and development to improve mood outcomes *via* the gut-brain axis (17). Animal studies have observed that probiotics improved depression by increasing monoamine neurotransmitters levels or by reducing proinflammatory cytokine levels (8, 18, 19). More importantly, studies have increasingly reported that the intestinal microbiota may influence the inflammatory responses and in so doing may modulate mood and depression-like behaviors (7, 20). *Lactobacillus rhamnosus* HN001 (HN001) and *Bifidobacterium animalis* subsp. *lactis* HN019 (HN019) have been shown to transiently colonize the gut microbiota, and in addition support immune function and reduce inflammatory activity (21–25). Moreover, previous research has indicated that HN001 could reduce postnatal depression and anxiety (26, 27). Thus, based on previous studies, we hypothesized that combined HN001 and HN019 supplementation could impact intestinal bacteria, and impact neurotransmitter and inflammatory cytokine levels to a greater extent than single supplementation with single strains, and therefore more effectively improve depression.

External chronic stress is a common risk factor for psychiatric disorders such as depression and anxiety, which can lead to permanent changes in the central nervous system (CNS) (28, 29). Chronic unpredictable mild stress (CUMS) is a rodent model of depression, which is currently the most commonly used and effective (30, 31). Meanwhile, a large variety of similar pathophysiological characteristics have been found in mood and anxiety disorders that co-occur in nearly 50–60% of clinical subjects (32). Indeed, animals exposed to chronic stress typically display clear-cut depressive behavioral deficits, such as anhedonia, helpless, cognitive impairment, as well as anxiety (33). Thus, in the present study, we employed the CUMS model to explore effects of HN001 and/or HN019 on the gut microbiota, neurotransmitters, inflammatory cytokines, and depression-like behaviors. The correlation between gut microbiota, neurotransmitters, and inflammatory factors was analyzed by informatics data analysis to explore the potential mechanism.

## Materials and methods

### Animals and groups

Two-month-old Male Sprague-Dawley (SD) rats (SPF Biotechnology Co, Ltd, Beijing, China) weighing 180–220 g were used to build CUMS model of depression. All rats were housed under a regulated environment: 12 h light/dark cycle, 25 ± 1°C temperature, 70 ± 2% humidity, free access to food and water. After adaption feeding, behavior tests and weight were measured to ensure rats baseline and each groups no difference in behavior. All rats were randomly classified into six groups by body weight and behavior in equal numbers. There was no significant difference in the baseline body weight and behavior among the groups. According to whether probiotics and positive medicine were added, the six groups were named as follows: non stressed group (control, *n* = 10), CUMS group (CUMS, *n* = 10), CUMS and *Lactobacillus rhamnosus* HN001 group (CUMS-HN001, *n* = 10), CUMS and *Bifidobacterium* lactis HN019 group (CUMS-HN019, *n* = 10), CUMS and HN001&HN019 group (CUMS-HN001&HN019, *n* = 10), CUMS and fluoxetine (CUMS-Flu, *n* = 9). The animal experiment was performed according to the National Institutes of Health Guide for the Care and Use of Laboratory Animals. Approvals for the study were acquired from the Animal Ethical and Welfare Committee of Tianjin Nankai Hospital.

### Establishment of chronic unpredictable mild stress rat model

Chronic unpredictable mild stress (CUMS) is one of the behavioral models leading to depression-related behaviors. Briefly, the stressed rats were exposed to nine stressors for 6 weeks, while control rats were normally kept in a non-stressful environment. Stressors included food deprivation 24 h, water deprivation 24 h, swimming in 4°C for 5–10 min, reversed light/dark cycle for 24 h, 60 Hz noise for 1 h, wet bedding for 8 h, tail pinching for 2 min, cage shaking for 15 min, physical restraint for 2 h. Two stressors were randomly applied each day to ensure unpredictability. Meanwhile, in order to avoid the adaptability of rats to stressors, there will be no repeated stressors within 3 days.

**Figure 1:**
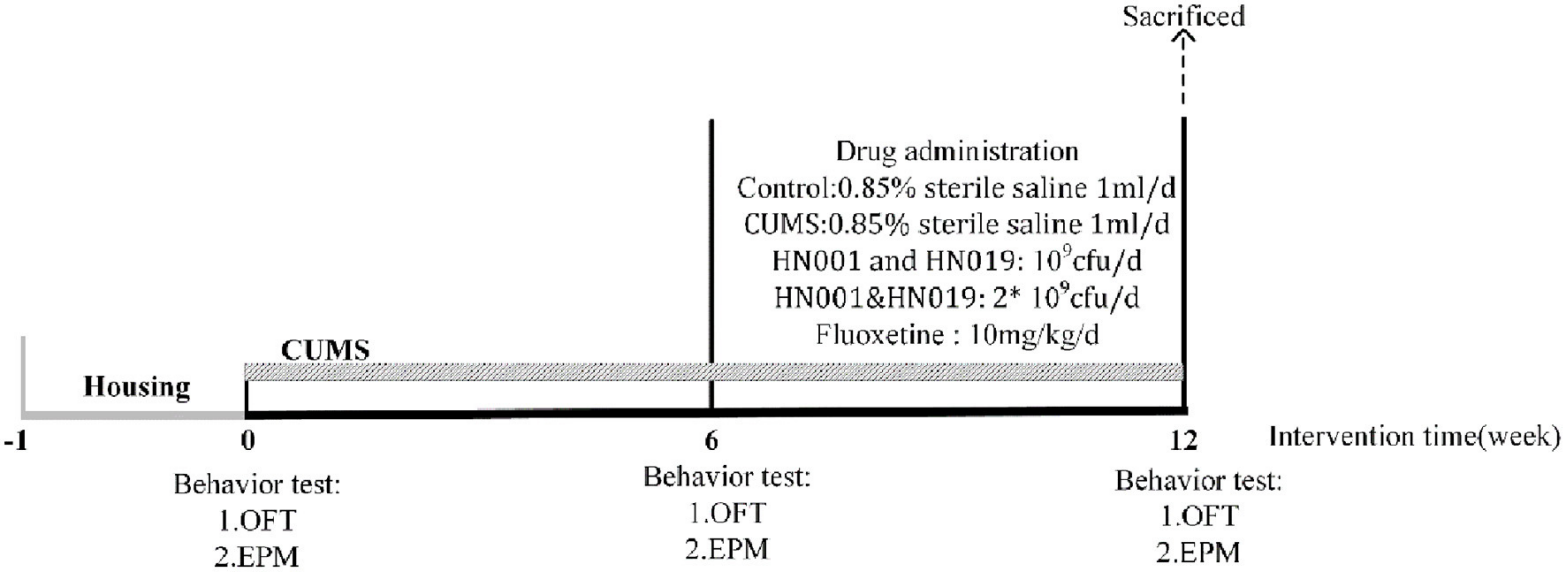
The entire experiment producer. CUMS chronic unpredictable mild stress, OFT open-field test, EPM elevated-plus test.

After adaption feeding, behavior tests were be tested to ensure rats baseline and each groups no difference in behavior. After stimulating lasting 6 weeks, compared with control group, other groups with CUMS have significant difference in behavioral experiments, so CUMS model was be successfully established. Then, they received treatment for the next 6 weeks within continued CUMS stimulation. Finally, behaviors of all intervention groups were significantly improved compare with the CUMS group, so the animals were sacrificed. A scheme of the experiment is shown in Figure 1.

After 6 weeks treatment, all rats which were euthanized with CO_2_ after fasting overnight were sacrificed. Blood was collected by aorta ventralis puncture and then was centrifuged at 3,000 rpm for 15 min at 4°C to obtain serum. Brain tissue and colon were either fixed with 4% (w/v) paraformaldehyde for histopathological staining or stored at −80°C after flash-freezing in liquid nitrogen for biochemical tests. Feces were collected in sterile tubes and then stored at −80°C.

### Treatments

Except the CUMS-Flu group, rats in other groups were given 0.85% saline orally (1 mL/d, control group and CUMS group) or with HN001 (10^9^CFU/d, CUMS-HN001 group) or with HN019 (10^9^CFU/d, CUMS-HN019 group) or with HN001 and HN019 (2 × 10^9^CFU/d, CUMS-HN001&HN019 group). The CUMS-Flu group received fluoxetine 10 mg/kg/d orally. Drug treatments were performed at the same time every day (9:00 am−11:00 am).

### Behavioral tests

#### Open-field test

Open-field test was performed in a black square box (62.5 × 74 × 51 cm, L × W × H). Rats were trained moderately early before the open field test. Experimental rats were placed in a fixed position in the center area and allowed to explore the area for 5 min. The total distance of movement and the number of standing times within 5 min of the rats were recorded and analyzed using the ANY-maze software to evaluate the exploratory and locomotor activity. During the experiment, the environment should be kept quiet and dim. Between subjects, the box was thoroughly cleaned with 75% alcohol and completely dried.

#### Elevated-plus maze test

The EPM apparatus consisted of two open arms (50 × 10 cm, L × W) across from each other, which were perpendicular to two closed arms (50 × 10 × 40 cm, L × W × H). Before the experiment, rats were trained moderately, then rats were placed in the center platform facing the same closed arm and allowed to freely explore for 5-mins, and exploratory activities in both open and closed arms were recorded and analyzed using the ANY maze software. During the period, the total distance of movement and the time of entries in the closed arms were recovered. This apparatus was cleaned with 75% alcohol and completely dried after occupancy by each rat.

### Physiological tests

#### Determination of inflammatory factors and neurotransmitters

Levels of inflammatory factors and neurotransmitters were measured by Enzyme-linked Immunosorbent Assay (ELISA) in serum and brain tissue, as well as in the colon. Centrifuge the whole blood sample collected in the serum separation tube at 3,000 × g for 15 min, and then stored the supernatant at −80°C. The tissues were raised with pre-cooled PBS (0.01M, pH = 7.4), and cut into pieces after weighing. Put the trimmed tissue into PBS solution (1:9 weight-volume ratio, and added protein inhibitor to PBS) for homogenization, finally centrifuge the homogenate at 5,000 × g for 10 min, and the supernatant was stored at −80°C. A bicinchoninic acid (BCA) Protein Assay kit (SparkJade, China) was used to determine the protein concentration. Levels of IL-6, IL-1β, IL-18 and TNF-α were determined using ELISA Assay kits (JiangLaibio, China) according to manufacturer's instructions. The concentration of 5-HT, DA, NE, HIAA, HVA and DOPAC were determined using the ELISA Assay kits purchased from Jiangsu Meimian Industrial Co, Ltd. (Jiangsu, China) as manufacturer instructions.

#### Histopathological analysis

The rats were deeply anesthetized and perfused with 4% paraformaldehyde in 0.1 M phosphate-buffered saline (PBS), then the brains and the colons were removed. For hematoxylin and eosin (HE) staining, the brain tissues and the colon tissues were fixed in 4% paraformaldehyde, embedded in paraffin and cut into 3 μm. The tissue sections were dewaxed with xylene (2 times; 5 min each) and hydrated with a series of decreasing concentrations of ethanol (95, 80, 70%, 2 min each) and in distilled water for 2 min. After staining with hematoxylin for 15 min, the sections were rinsed with tap water. Then the sections were incubated in acidic liquid alcohol for 30 s, immersed in tap water for 15 min, then stained with eosin for 5 min and dehydrated by immersing in gradient ethanol (95, 95, 100, 100%) for 2 s and xylene twice for 1 min. Finally removed with xylene and fixed with neutral resin. Additionally, the sections were observed under inverted microscope. For Nissl staining, the sections were immersed in xylene for 30 min and were then gradually hydrated by immersion in a series of decreasing concentrations of ethanol (100, 90, 75, and 50%, 2 min each) and in distilled water for 2 min. Then the tissues sections were stained with Nissl dye liquor (heated in an oven at 37°C) for 5 min and rinsed quickly in distilled water. Finally, the tissues sections were cover-slipped with PBS and visualized under inverted microscope.

#### 16S rRNA sequencing

The fecal samples were taken from seven rats in each group after intervention, and Fecal DNA was extracted by using HiPure Soil DNA Kit (Magen, Guangzhou, China). The 341F/806R primer set (341F: CCTACGGGNGGCWGCAG, 806R: GGACTACHVGGGTATCTAAT) was used to targeting the V3-V4 region. The collected amplicons were purified using the AxyPrep DNA gel extraction kit (Axygen Biosciences, Union City, CA, USA), and the ABI StepOnePlus real-time PCR system (Life Technologies, Foster City, USA) was used for quantification. Illumina sequencing platform was used to sequence the PCR products in the 16S rRNA V3–V4 region to validate the differences of intestinal flora among groups. And Usearch method was used to process the data to obtain OTU representative sequence and OTU abundance information. Based on OTU and species abundance tables, carried out biological information analysis such as species composition, Alpha diversity, Beta diversity.

### Statistical analysis

SPSS version 24.0 was used to conduct the statistical analyses. All data were presented as Mean ± SEM, and the statistical analyses among groups were performed by using one-way ANOVA with LSD *t*-test for comparison between every two groups. Results were regarded as statistically significant at *p* < 0.05. Sequences analysis were performed by UPARSE software package using the UPARSE-OTU and UPARSE-OTUref algorithms. We rarified the OTU table and calculate four metrics: Chao1 index, ACE index, Sobs index and Shannon index to compute Alpha diversity. PCoA based on weighted and unweighted UniFrac metrics was used to assess the variation of bacterial composition among different groups. To identify differences of microbial communities between the different groups, ANOSIM and ADONIS were performed based on the Bray-Curtis dissimilarity distance matrices. To confirm differences in the abundances of individual taxonomy between the different groups, STAMP software was utilized. LEfSe was used for the quantitative analysis of biomarkers within different groups. The relative abundance of fecal microbiota in the six groups was compared using the Kruskal–Wallis H test. Spearman's rank correlation analysis was further employed to assess the associations among the microbiota inflammatory factors and neurotransmitters. A *p*-value <0.1 was considered to be statistically significant.

## Results

### Body weight and depression-like behavior

**Figure 2:**
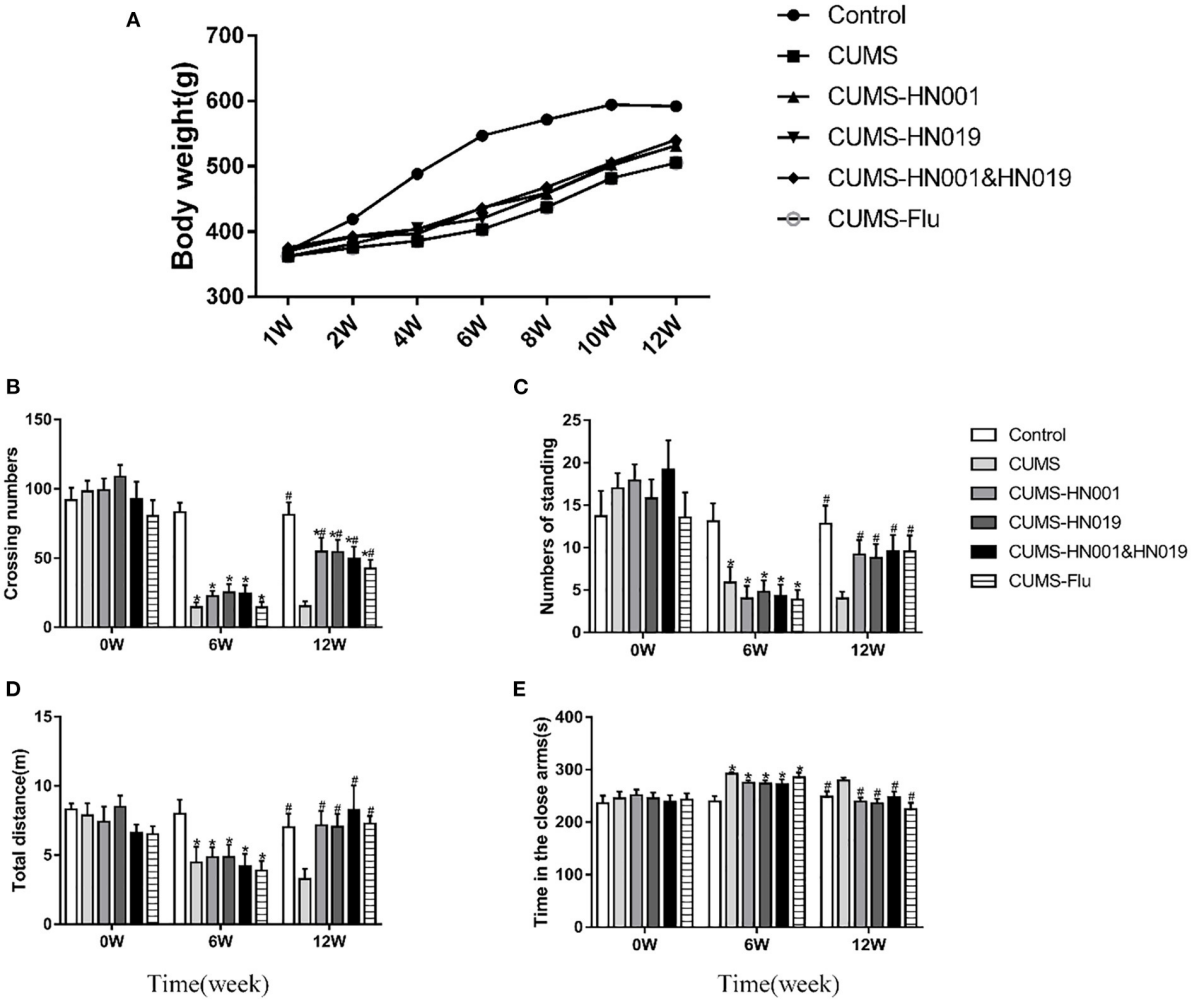
Body weight and depression-like behavior of CUMS rats in different groups during the experiment. **(A)** Changes of body weight of rat under CUMS. **(B)** Crossing numbers in open-field test during baseline, 6th and 12th weeks. **(C)** Numbers of standing in open-field test during baseline, 6th and 12th weeks. **(D)** Total distance in elevated-plus during baseline, 6th and 12th weeks. **(E)** Time in the close arms in elevated-plus during baseline, 6 and 12th weeks. Data are expressed as Mean ± SEM (*n* = 9–10). *p < 0.05 compared with control group. #*p* < 0.05 compared with CUMS group.

The control group rats gained weight normally during the 12-week experiment. In contrast, CUMS stimulation decreased the rate of weight gain, which was improved by probiotic supplementation (Figure 2A).

In the open-field test, there were no significant differences in the number of crossing lines and standing times between the groups at baseline (*p* > 0.05). However, following CUMS treatment, these two indicators of open-field behaviors were prominently reduced compared to control group (*p* < 0.05). Compared with CUMS group, crossing numbers and standing times in CUMS-HN001group, CUMS-HN019 group and CUMS-HN001&HN019 group were significantly increased (*p* < 0.05) (Figures 2B,C).

In the elevated-plus test, CUMS-treated rats administrated HN001 and/or HN019 spent less time in the closed arm and traveled a significantly greater distance compared to the CUMS-only rats (*p* < 0.05) (Figures 2D,E).

Together, these results suggested that rats exposed to CUMS showed depressive-like behaviors in both the open field test and the elevated plus maze, and that treatment with HN001 and/or HN019 could attenuate the induction of depression-like behaviors. However, for both behavioral tests, there were no significant difference between the combined probiotics and each single probiotic.

### Probiotics normalized the morphological changes of hippocampi and colon

**Figure 3:**
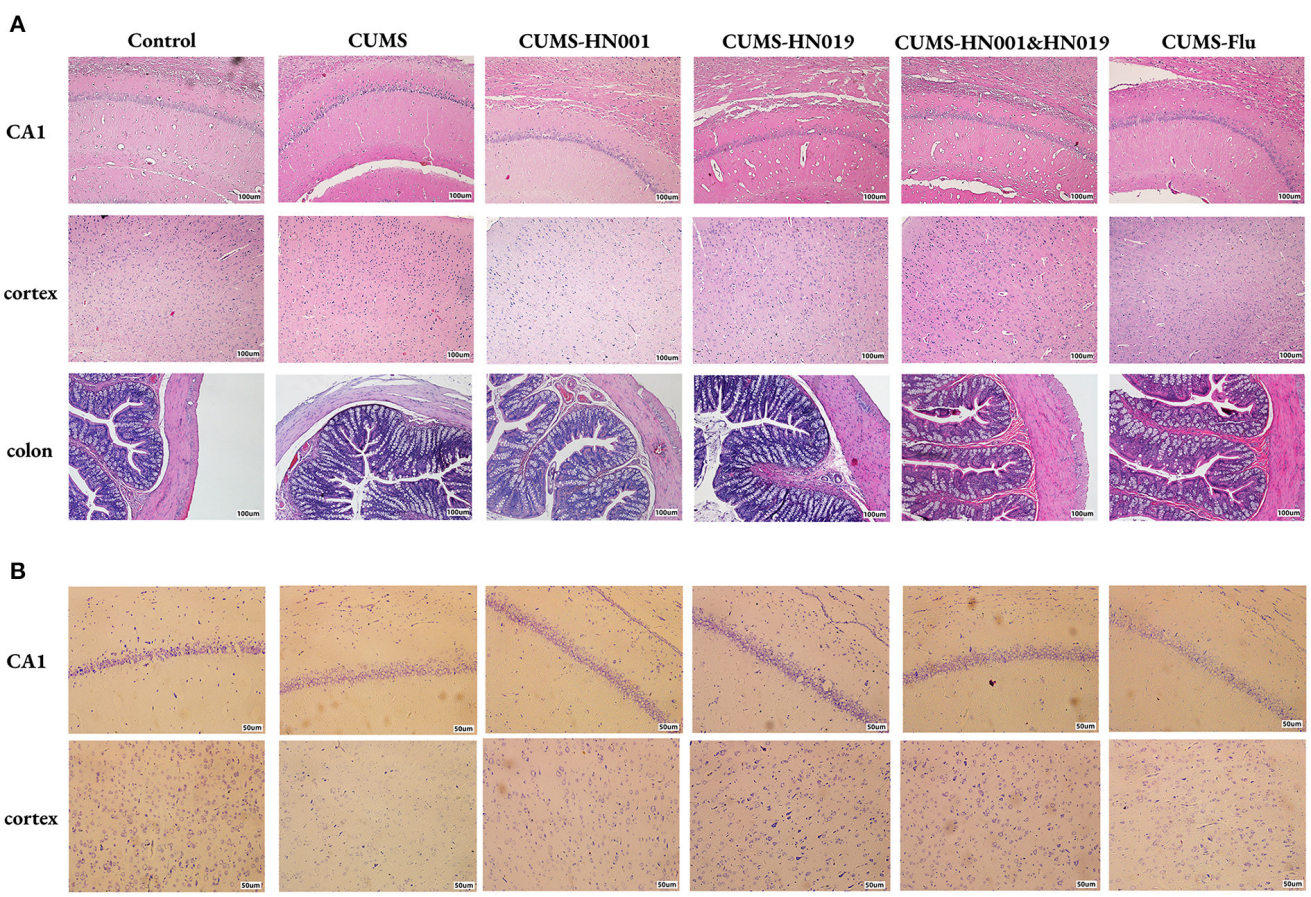
Probiotics supplementation improved the histological variations in rat hippocampi and colon. Representative micrographs of hippocampus, cortex and colon labeled with HE staining **(A)**. Scale bar = 100 μm. Neurons in hippocampus and cortex were assessed by Nissl staining **(B)**. Scale bar = 50 μm (*n* = 3 brains/group).

As shown in Figure 3A, the hippocampal CA1 region and cortex of control group rats showed numerous, morphologically normal and compactly arranged neurons. In contrast, CUMS-treated rats displayed irregularly arrayed neurons, nuclei shrinkage, and unclear nucleoli. Similarly, compared to control rats, the intestinal villi of the CUMS rats were disordered and obviously damaged, with reduced thickness of the intestinal mucosa. However, tissues from CUMS-treated rats administered HN001 and/or HN019 showed significant reduction in these signs of histopathological damage. Nissl staining was used to examine neuronal morphology (Figure 3B). The numbers of Nissl bodies were significantly increased in non-CUMS control rats and CUMS rats treated with HN001 and/or HN019 compared with CUMS rats not supplemented with probiotics.

### Effects of probiotics on neurotransmitters and their metabolites in serum and brain

**Figure 4:**
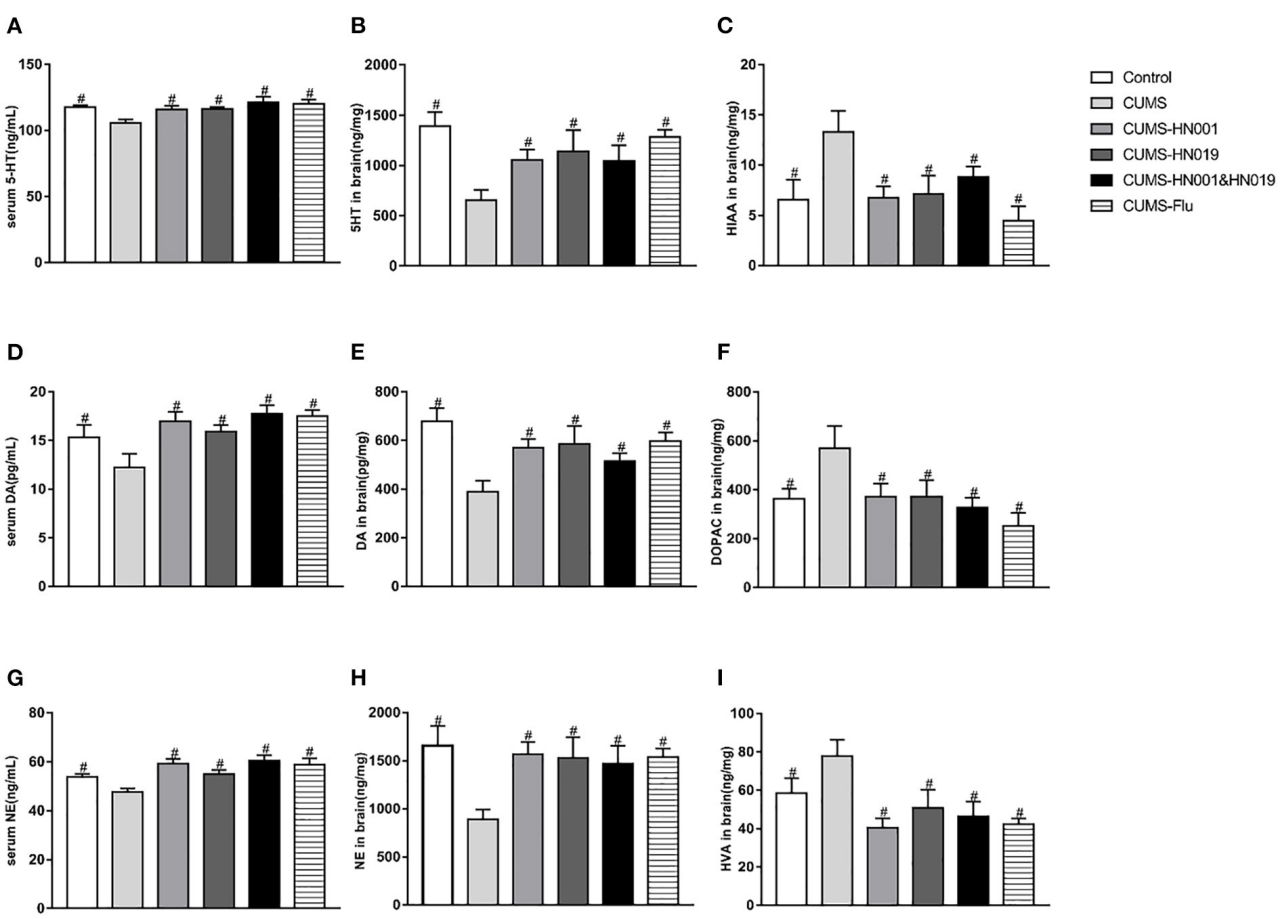
Probiotics normalized CUMS-induced changes in neurotransmitters and their metabolite in rat serum and brain. The level of serum and brain 5-HT, DA and NE were detected at the end of the experiment **(A,B,D,E,G,H)**. **(C**,**F**,**I)** showing metabolite of monoamine neurotransmitters in brain. The results are presented as the Mean ± SEM (*n* = 9-10 in serum, *n* = 6 in brain). #*p* < 0.05 compared with CUMS group.

CUMS significantly decreased the content of 5-hydroxytryptamine (5-HT), dopamine (DA), norepinephrine (NE) in serum and brain (*p* < 0.05) (Figure 4). 5-hyroxyindole acetic acid (5-HIAA), dihydroxyphenylacetic acid (DOPAC) and homovanillic acid (HVA), as the metabolite of 5-HT, DA and NE were all increased in rat brain samples after CUMS stimulation. Administration with HN001 and/or HN019 was able to reverse the effects of CUMS on these neurotransmitters (*p* < 0.05). However, there was no significant difference between HN001, HN019, or their combination.

### Probiotics attenuated elevated inflammatory factors in serum, brain and colon

**Figure 5:**
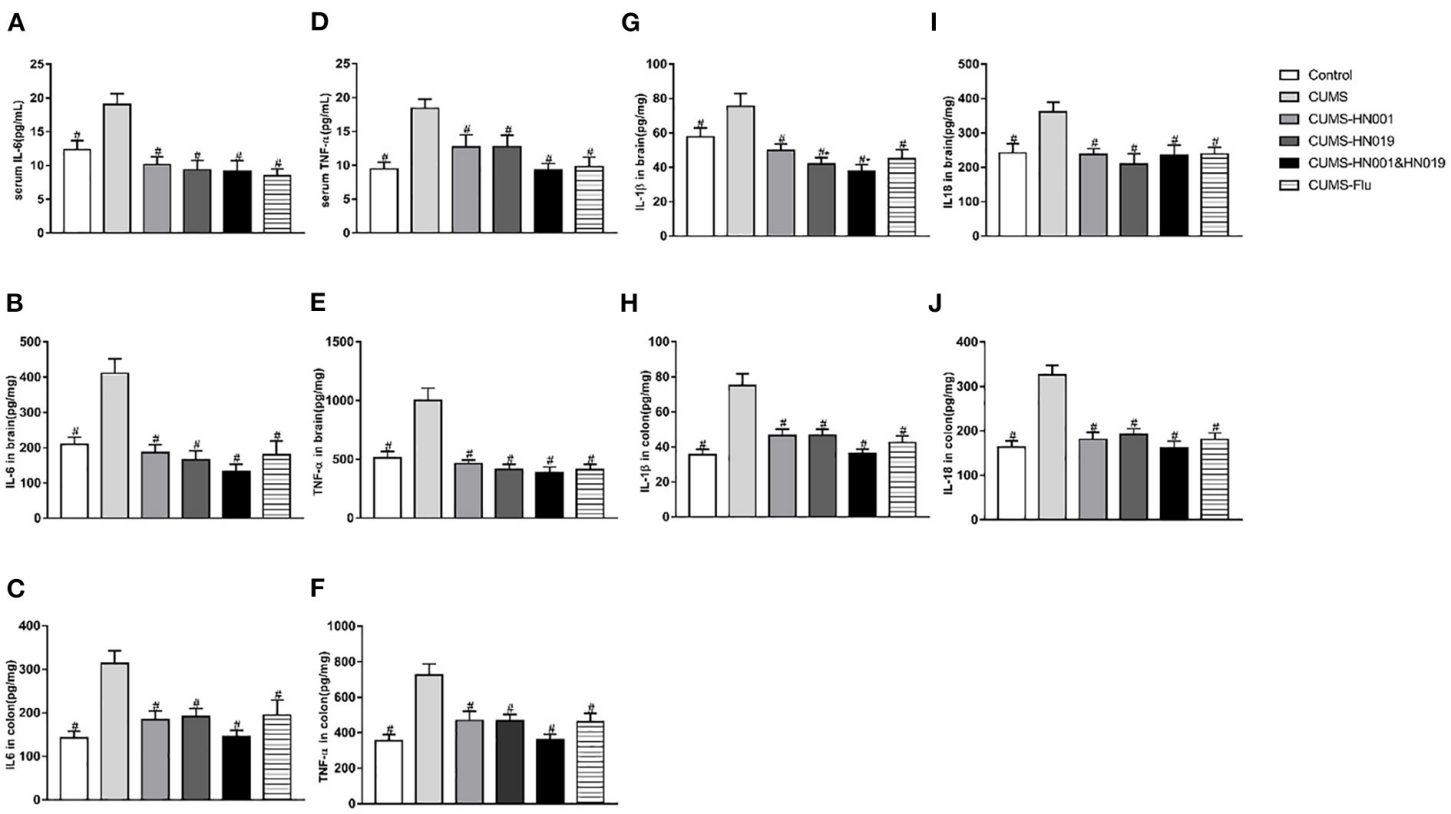
The levels of IL-6, IL-18, IL-1β and TNF-α in serum, brain and colon of CUMS rats in different groups during experiment. These indicators were tested at the end of experiment. The results were presented as the Mean ± SEM (simple in serum was 9-10, simple in brain and colon was 6). *p < 0.05 compared with control group. #*p* < 0.05 compared with CUMS group.

Following CUMS treatment, concentrations of IL-6, TNF-α, IL-1β, and IL-18 in serum, brain, and colon were significantly elevated compared with controls (*p* < 0.05). The application of HN001 and/or HN019 treatment suppressed the CUMS-dependent rise in these inflammatory factors. No significant differences in inflammatory factor levels in serum, brain and colon were found between the three intervention groups (Figure 5).

### Correlation analysis of inflammatory factors in brain and colon

**Figure 6:**
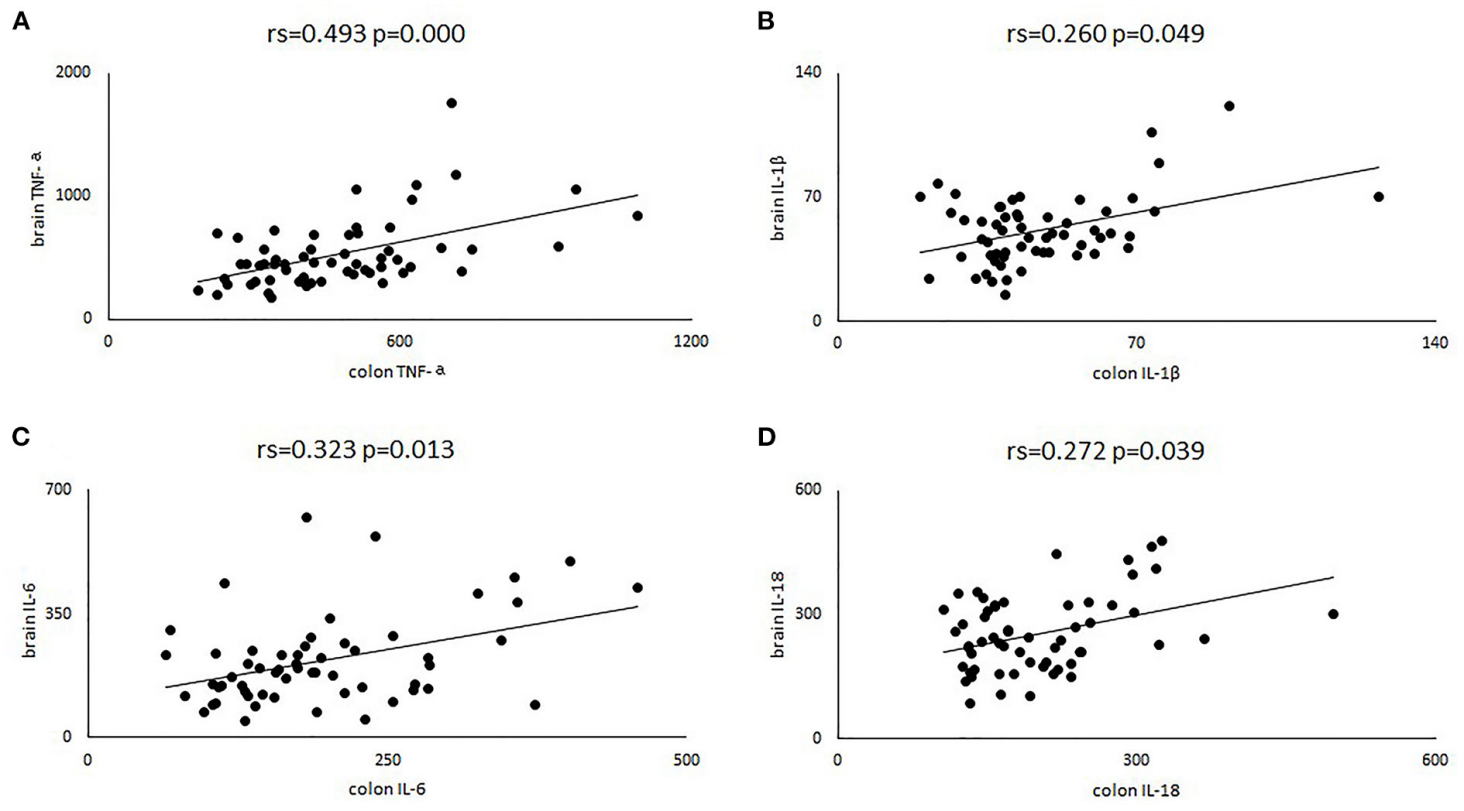
Four scatter plots were used to describe the correlation of TNF-α **(A)**, IL-6 **(B)**, IL-1β **(C)** and IL-18 **(D)** in brain and colon. Rs, represent the spearman correlation coefficient.

To further verify that intestinal inflammation was linked to brain inflammation, we assessed the correlation between intestinal inflammatory factors and those in the brain. We found that TNF-α in the brain and colon showed a clear positive correlation (rs = 0.493, *p* = 0.000). Positive correlations were also observed for IL-6 (rs = 0.323, *p* = 0.013), IL-1β (rs = 0.260, *p* = 0.049) and IL-18 (rs = 0.272, *p* = 0.039) between the brain and colon (Figure 6).

### Alterations of microbiota induced by probiotics treatment

**Figure 7:**
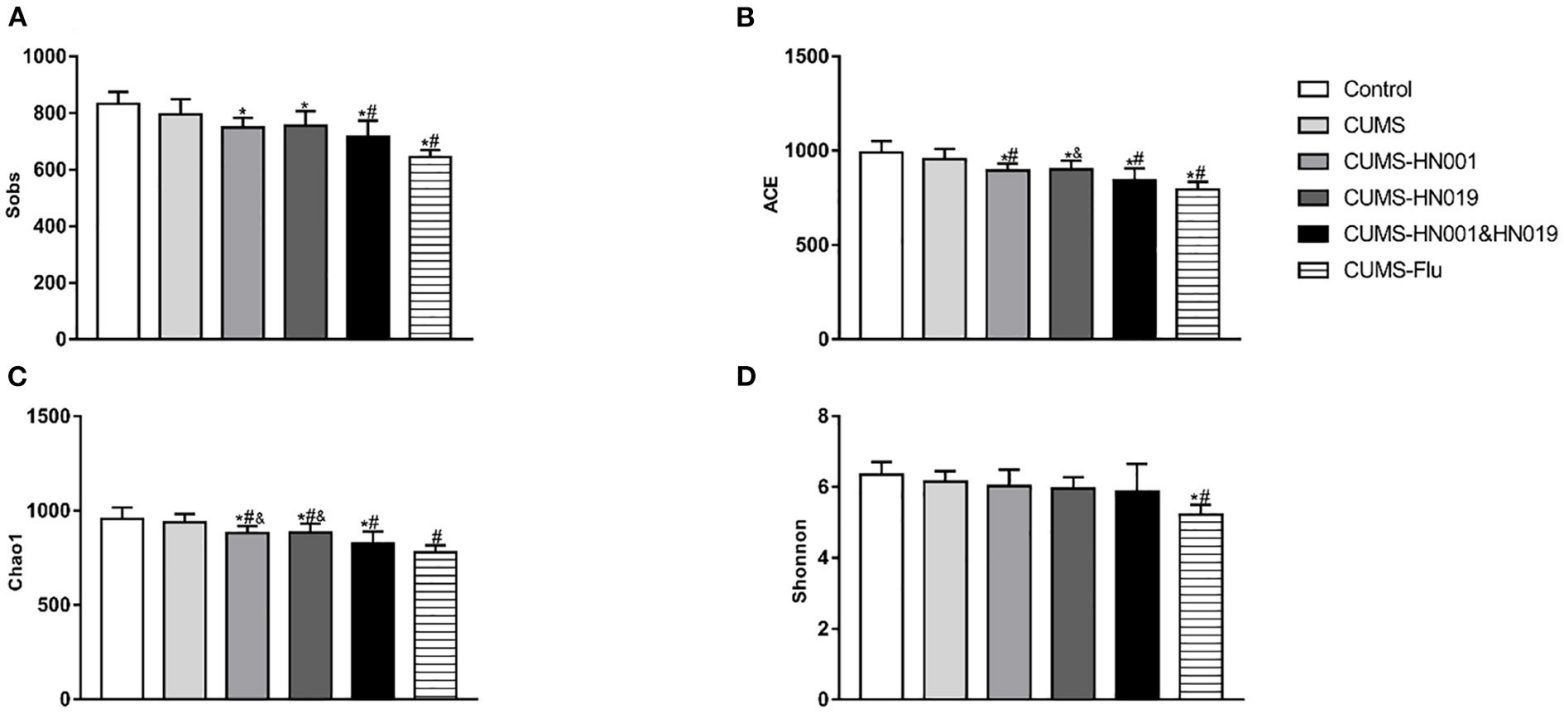
Effects of probiotics treatment on gut microbiota changes induced by CUMS. **(A)** Sobs index, **(B)** ACE index, **(C)** chao1 index, **(D)** Shonnnon index. The results were presented as the Mean ± SEM (*n* = 7). *p < 0.05 compared with control group. #*p* < 0.05 compared with CUMS group, &*p* < 0.05 compared with CUMS-HN001&HN019 group.

To evaluate whether CUMS treatment could alter intestinal microbiota, 16S rRNA sequencing was performed fecal samples. While alpha-diversity, as measured by richness (Sobs index, Chao1index and ACE index), and diversity (Shannon index) decreased after CUMS treatment, differences compared with control group animals were not statistically significant (Control-CUMS: *p* > *0.05*). Interestingly, compared with CUMS group animals, ACE and Chao1 index significantly decreased in CUMS groups administrated HN001 and/or HN019 (CUMS-CUMS-HN001: *p* < *0.05*, CUMS-CUMS-HN019: *p* < *0.05*, CUMS-CUMS-HN001&HN019: *p* < *0.05*). Moreover, the Chao1 Index in the HN001&HN019 was significantly decreased compared with that of HN001 or HN019 groups (CUMS-HN001-CUMS-HN001&HN019: *p* < *0.05*, CUMS-HN019-CUMS-HN001&HN019: *p* < *0.05)*, which suggested marked change in the diversity of intestinal microbiota. As changes in the dominant intestinal microbiota after probiotic intervention may be responsible, further analyses are required to find out whether dominant flora may be related to anxiety and depression-like behaviors (Figure 7 and Supplementary Figure 1).

**Figure 8:**
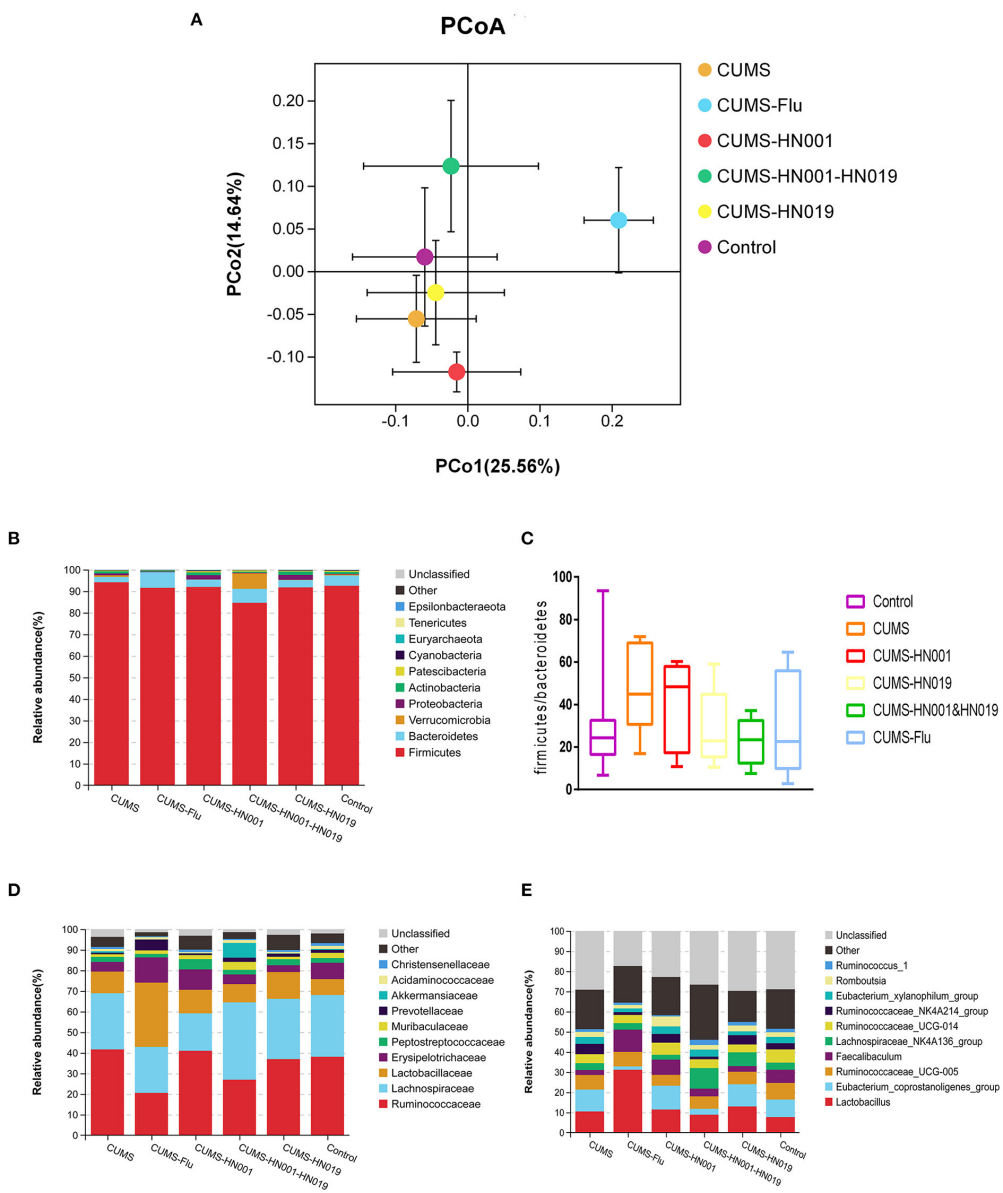
Analysis of microbiota flora composition among six groups. **(A)** unweighted PcoA, **(B,D,E)** difference of microbiota flora abundance at the phylum, family and genus among six groups. The area of each color represents the abundance of different bacteria in the intestine. The larger the area, the higher the abundance, and vice versa. Data are stated as mean percentage values from each group (*n* = 7). **(C)** Comparison of the ratio of Firmicutes to Bacteroidetes among six groups.

Beta diversity analysis was performed to assess differences in microbial composition between the six experimental groups by principal component analysis (PCoA) (Figure 8A and Supplementary Figure 2). The PCoA results showed that there was no difference in microbial communities among the different groups (PERMANOVA: Control-CUMS: *F* = 1.414, *p* = 0.179, CUMS-CUMS-HN001: *F* = 1.343, *p* = 0.160, CUMS-CUMS-HN019: *F* = 0.729, *p* = 0.723). Moreover, CUMS rats treated with HN001&HN019 showed marked differences in PCoA plots compared to CUMS rats with single probiotic treatment (CUMS-CUMS-HN001&HN019: *F* = 2.609, *p* = 0.001, CUMS-HN001-CUMS-HN001&HN019: *F* = 3.941, *p* = 0.001, CUMS-HN019-CUMS-HN001&HN019: *F* = 2.306, *p* = 0.001). Furthermore, ANOSIM also confirmed shorter distance among different groups (*R* = 0.443, *p* = 0.001, Supplementary Figure 2). In addition, there was significant difference between treatment groups (betadisper: *F* = 2.867, *p* = 0.001). These results suggested that combined probiotics could significantly change microbiota compared to single probiotics.

According to the species abundance cluster heatmap (Figure 8B), at the phylum level Firmicutes, Bacteroidetes, Verrucomicrobias, Proteobacterias and Actinobacterias were dominant. Relative abundances are shown in Supplementary Figure 3. CUMS-only treatment down regulated the relative abundance of Bacteroidetes, but upregulated the relative abundance of the other bacterial phyla mentioned above. Treatment with HN001 and/or HN019 alleviated the phylum alterations, with the changes associated with HN001&HN019 more marked. While the Firmicute to Bacteroidetes ratio (*F*/*B* ratio) increased following CUMS stimulation, the administration with HN001 and/or HN019 tended to decrease the F/B ratio, which was consistent with previous study (34), but did not reach statistical significance difference (*p* > 0.05).

At the family level, the relative abundances of Ruminococcaceae and Lactobacillaceae were elevated, while the Lachnospiraceae declined in the CUMS-only group. Administration with HN001 and (or) HN019 reversed these CUMS-related changes, especially so for HN001&HN019 group. The genera Lactobacillus, Lachnospiraceae-NK4A136-group, and Eubacterium_coprostanoligenes_group, all of which belong to the Firmicutes phylum, were altered by CUMS, and these changes were also improved by HN001 and/or HN019 supplementation. Although relative ratios of microbiota were changed after CUMS treatment and by HN001 and/or HN019 interventions, the difference between the six experimental groups were not significant.

### Correlation analysis of intestinal microbiota with monoamine neurotransmitters and inflammatory factors

**Figure 9:**
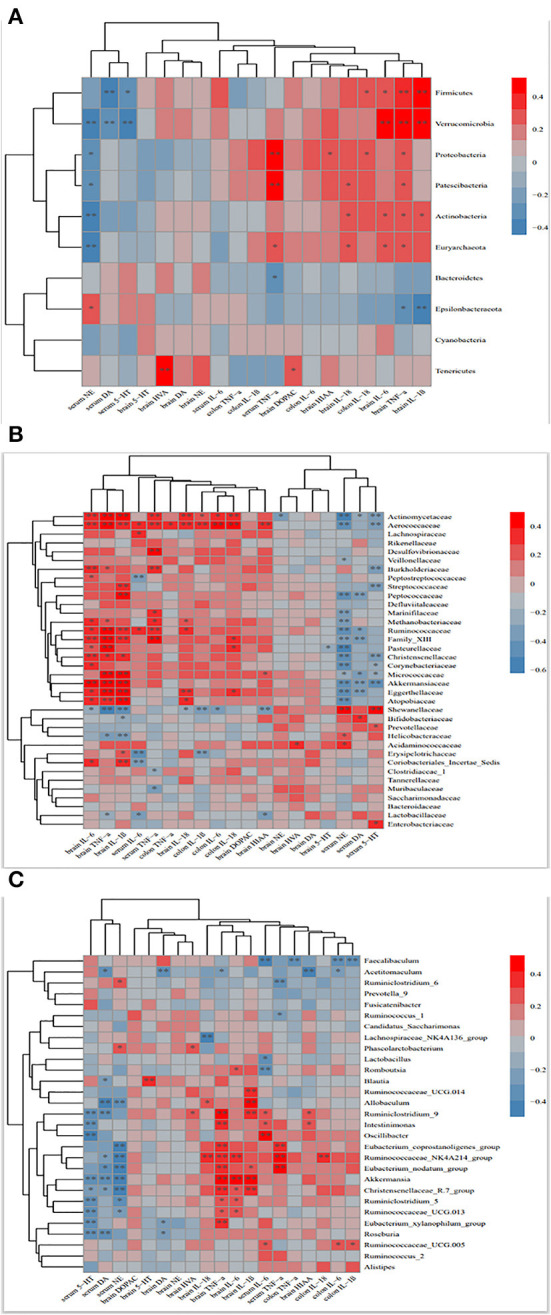
Heat map showing the correlation between the phylum **(A)**, family **(B)**, genus **(C)** and monoamine neurotransmitters and Inflammatory factors. Red and blue colors indicate positive and negative correlations, respectively. *p <0.1, **p < 0.05.

Monoamine neurotransmitter deficiency and inflammatory factor imbalance are important factors in the pathogenesis of depression (35, 36). To elucidate possible associations between altered microbiota and depression-related traits such as neurotransmitters and inflammatory factors, Spearman's correlation analysis was performed. As shown in Figure 9, Firmicutes and Verrucomicrobia was positive association with brain TNF-α (rs = 0.324, *p* = 0.037, rs = 0.519, *p* = 0.065) and IL-1β (rs = 0.408, *p* = 0.007, rs = 0.472, *p* = 0.065), respectively. Proteobacteria, Pastescibacteria, Actinobacteria and Euryarchaeota showed positive correlations with inflammatory factors in serum, brain and colon, and negative correlations with serum levels of NE, DA and 5-HT. In contrast, Epsilonbacteraeota and Bacteroidetes showed positive correlations with serum neurotransmitter concentrations, and negative correlations with inflammatory mediators. Other correlations could be identified between the family and genera microbiota and these indicators. In particular, family Lachnospiraceae, Ruminococcaceae, Christensenellaceae and Peptostreptococ-caceae and genera Ruminococcaceae, Eubacterium_coprostanoligenes_group, Ruminococcaceae_UCG014, Ruminoco-ccaceae_NK4A214_group and Ruminococcus-1 (all of which belong to the Firmicutes phylum), showed positive and negative associations with inflammatory factors and serum neurotransmitters, respectively. Muribaculaceae and Prevotellaceae (Bacteroidetes phylum) were negatively related with inflammatory factors and positive correlation with serum 5-HT, NE, and DA, which were consistent with Bacteroidetes. These results indicated that altered microbiota, especially certain microbiota, which were specifically affected by HN001 and (or) HN019, showed strong associations with neurotransmitters and inflammatory cytokines simultaneously, demonstrating the critical role of gut microbiota in regulating neurotransmitters and inflammation.

## Discussion

Psychobiotics were bacteria that, when ingested in adequate amounts, could produce a positive mental health benefit for patients suffering psychiatric illness (37). Both animal and human studies have shown that psychobiotics, such as Bifidobacterium infantis and Bifidobacterium longum NCC3001, may play roles in preventing depression-like or anxiolytic-like behaviors (38, 39), suggesting that probiotics might be a potential therapeutic for depression. Usually, the effective dose range is 10^7^-10^11^CFU (colony forming units) per day. At present, the recommended intake of probiotics in the research literature and relevant international standards and regulations is mainly 10^7^-10^11^CFU/d. The vast majority of studies in the literature included a dose of 10^9^ CFU/d (40, 41). The results of our current study suggested that probiotics HN001 and (or) HN019 may also have psychobiotic activity. Our results demonstrated that intervention with HN001 and/or HN019 improved the depression-like and anxiety-like behaviors in CUMS-treated rats as by the open field test and the elevated plus maze. Also, probiotics intervention was able to reverse many of the neurotransmitter, inflammatory factor, and gut or brain morphological changes associated with CUMS treatment.

Previous studies have indicated that the microbiota might play a significant role in major depressive disorders through the microbiome-gut-brain axis (42, 43). In addition, intestinal microbiota alterations induced by providing probiotics and prebiotics could effectively improve the depression-like behaviors (44, 45). In the present study, we found that microbiota abundance and diversity were unchanged after 6 weeks of CUMS treatment, consistent with previous studies (46, 47). However, ACE index and chao1 index, which represent microbial richness and evenness, decreased more significantly after treatment with both HN001&HN019 in CUMS rats, suggesting that the abundance and diversity in the combined probiotic group changed markedly.

However, findings regarding the diversity and abundance of microbiota flora in depression and anxiety remain inconsistent. For example, Jiang et al. (10) and Kelly et al. (48) reported an increase in fecal bacterial diversity and abundance associated with depression, whereas both Naseribafrouei et al. (49) and Zheng et al. (46) observed no significant alterations in humans with depression. Such disparities may be due to a range of causes, such as differences in diets, modeling methods of depression, and type of sequence analysis method used.

In our study, intervention with CUMS and HN001and (or) HN019 induced 15 phyla, 58 families and 145 different genera alteration. Consistent with previous studies (47, 50), the abundance of Firmicutes, Actinobacteria, Proteobacteria and Lactobacillus increased, while the abundance of Bacteroidetes and Erysipelotrichaceae decreased in CUMS group compared with control group. Additionally, both increased Firmicutes and decreased Bacteroidetes abundance were associated with irritable bowel syndrome (IBS) (51). An increasing Firmicutes/Bacteroidetes ratio was related to several neurological disease (51–54) and inflammatory bowel diseases (55). Therefore, a reduced ration Firmicutes/Bacteroidetes (*F*/*B*) may be a potential index for the improvement of neurological condition. Administration of HN001&HN019 showed a marked effect in reducing the CUMS-induced changes in gut microbiota composition and decreasing the F/B ratio compared with single probiotics.

In addition, alterations of other bacteria in phylum, family and genus were observed and intervention with HN001&HN019 significantly ameliorate these changes. Based on the alterations in these gut microbiomes, the antidepressant effect of combined probiotics is more significant than that of using it alone. However, in this study, the characteristic gut microbiome induced by CUMS was not completely consistent with that found in previous studies. This difference may be due to breeding environment and modeling methods.

Depression is caused by a lack of monoamine neurotransmitters such as 5-HT, DA, and NE, and antidepressant drugs have therapeutic effects by increasing the monoamines neurotransmitters levels (56). Gut microbiota could synthesize part of neurotransmitters (57). Previous studies indicated that intervention with probiotics could improve depression by reducing 5-HIAA and DOPAC concentration and increasing the 5-HT level (19). In this study, we suggested that HN001 and (or) HN019 supplementation can alleviate the depression-like behaviors of rats by increasing the concentrations of neurotransmitters and decreasing content of neurotransmitter metabolites. Meanwhile, the effect of HN001 and HN001&HN019 on increasing DA and NE levels, and decreasing HIAA and DOPAC levels was superior to the HN019, which indicated that HN001 possibly played a key role in improving neurotransmitters. Our results found that phylum Epsilonbacteraeota and Bacteroidetes, as well as family Muribaculaceae and Prevotellaceae, which all of belong to Bacteroidetes were significantly positively associated with 5-HT, DA and NE. Additionally, correlations between other neurotransmitters and differential bacteria taxa were demonstrated in this study. Thus, these results further demonstrated that gut microbiota could regulate neurotransmitters levels. Due to the effects of combined probiotics in improving gut microbiota was superior to the single probiotics, so the impact of combined probiotics in increasing part neurotransmitters was more marked. However, correlations between microbiota and neurotransmitter were not enough consistent, and other factors such as inflammation and HPA axis could influence levels of neurotransmitters, so there was no significant difference between combined probiotics and single probiotics. Probiotics could influence the neurotransmitter receptor expression (58), so further researches are required to explore the changes of neurotransmitter, neurotransmitter metabolites and neurotransmitter receptor to verify whether the effect of combined probiotics is more marked than single probiotics.

Studies have demonstrated a strong relationship between depression and inflammatory response. Patients with depression have elevated inflammatory factors in peripheral blood, which could access the brain and interact with depression (59).

Consistent with the published data, we found that the levels of proinflammatory cytokines were increased in serum and brain of CUMS-induced rats. Meanwhile, levels of pro-inflammatory cytokines in the serum and brain of CUMS rats were increased, which was consistent with the published data. In addition, elevated cytokines and histopathological damage in the colon were observed in this study. When the level of intestinal inflammation was elevated, the intestinal mucosal barrier was damaged, thus promoting the entry of endotoxins and inflammatory cytokines into the blood system (60), which was evidenced by the increased serum cytokines in this study. In this study, these results suggested that HN00 and (or) HN019 intervention could decrease the concentrations of inflammatory cytokines, and the effects of combined probiotics in decreasing levels of IL-6, TNF-α, IL-1β and IL-18 was superior to single probiotics, which indicated that combined probiotics could more effective on decreasing the inflammation level. Recently, accumulating studies indicated that intestinal microbiota was associated with inflammation. Previous studies found that gut microbiota could impact inflammation by influencing levels of inflammation factors and producing Chain fatty acids (SCFAs), as well as changing intestinal barrier (61–63). Our results indicated that Firmicutes, Proteobacteria and Actinobacteria were significantly positively related with inflammatory factors, while the Bacteroidetes were negatively related with inflammatory factors. In addition, other differential bacteria taxa in phylum, family and genus were significantly related with inflammatory factors in this study. These results further demonstrated that gut microbiota could influence brain function by regulating of inflammation. Moreover, Administration with combined probiotics down-regulated the relative abundance of Firmicutes, Proteobacteria and Actinobacteria and upregulated Bacteroidetes, as well as changed other gut microbiota was more marked than single probiotics. Thus, the effect of combined probiotics on decreasing the inflammatory factors levels was superior to the single probiotics. However, there was no significant difference between single probiotics group and combined probiotics group. The reason may be due to the sample size and individuals' differences. Further studies are required to elucidate whether the effects of combined probiotics were more marked than single probiotics.

In conclusion, combined probiotics significantly ameliorated depression-like and anxiety-like behavior, possibly by up-regulating the expression levels of monoamines neurotransmitters, down-regulating levels of inflammatory cytokines, and amending structural changes of the intestinal microbiota of rats. Moreover, effects of combined probiotics on gut microbiota and inflammatory factors were more effective than single probiotics. Therefore, we suggest that development of interventional preparations mixed with probiotics may be a new method for the prevention and treatment of depression.

## Data availability statement

The raw data supporting the conclusions of this article will be made available by the authors, without undue reservation.

## Ethics statement

The animal study was reviewed and approved by the Animal Ethical and Welfare Committee of Tianjin Nankai Hospital.

## Author contributions

HL and XZe designed the research (project conception, development of overall research plan, and study oversight). LH and XL conducted the research (conduct of the experiment and data collection). ZM, JF, BS, MZ, and FW analyzed the data or performed the statistical analysis. LH, XL, and HL wrote the manuscript. HL had primary responsibility for the final content. All authors have read and approved the final version.

## Funding

This work was supported by Nutritional science research foundation from BY-HEALTH (No. TY202002002), the Scientific Research Project of Tianjin Educational Committee (No. 2019ZD026) and National Natural Science Foundation of China (Grant No. 82173516). The intervention used in the study were sourced from Fonterra Co-operative Group, New Zealand.

## Conflict of interest

Authors XZe, XZh, and RH were employed by BYHEALTH Institute of Nutrition & Health. The remaining authors declare that the research was conducted in the absence of any commercial or financial relationships that could be construed as a potential conflict of interest.

## Publisher's note

All claims expressed in this article are solely those of the authors and do not necessarily represent those of their affiliated organizations, or those of the publisher, the editors and the reviewers. Any product that may be evaluated in this article, or claim that may be made by its manufacturer, is not guaranteed or endorsed by the publisher.

## Supplementary material

Supplementary Figure 1 Effects of probiotics treatment on alpha-diversity induced by CUMS.

Supplementary Figure 2 Effects of probiotics treatment on Beta-diversity induced by CUMS. **(A)** Number of weight Beta-diversity estimates of microbiota. **(B)** The results of ANOSIM analysis based on Bray-Curtis distance.

Supplementary Figure 3 Relative abundances of selected microbes at the genus level. Data are expressed as Mean ± SEM (*n* = 7). ^*^*p* < 0.05 compared with control group. #*p* < 0.05 compared with CUMS group, &*p* < 0.05 compared with CUMS-HN001&HN019 group.
