# Supplementary figures and images for: Combined probiotics attenuate chronic unpredictable mild stress-induced depressive-like and anxiety-like behaviors in rats
Source: Front Psychiatry. 2022 Sep 7;13:990465. doi: 10.3389/fpsyt.2022.990465 (PMC9490273; doi:10.3389/fpsyt.2022.990465)

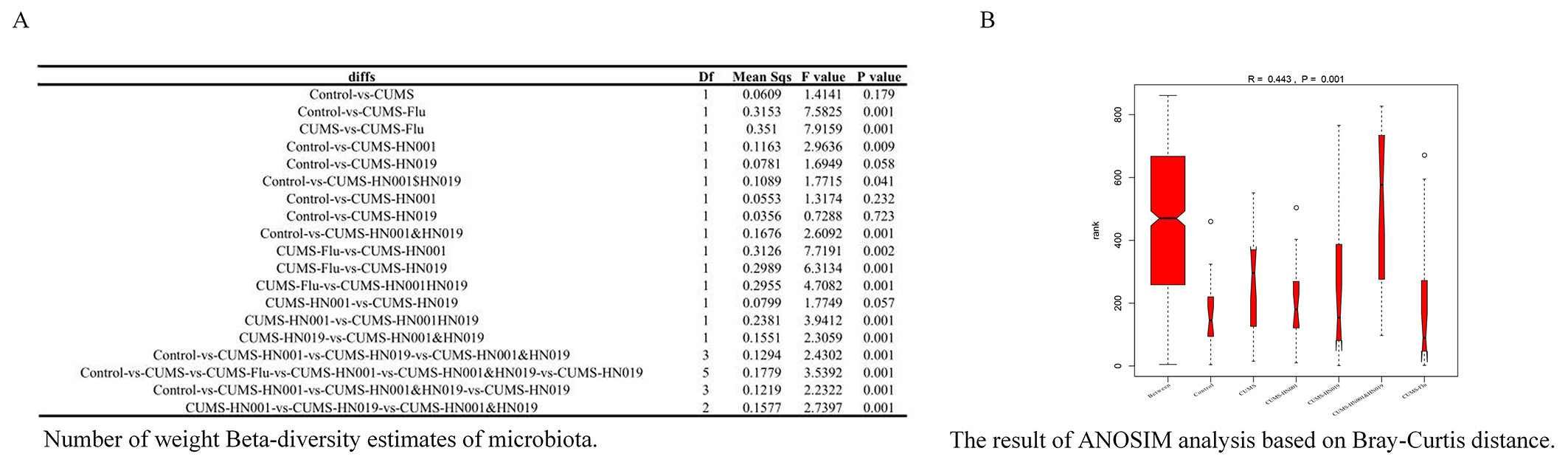

Supplement: Supplementary Figure 2 — Effects of probiotics treatment on Beta-diversity induced by CUMS. (A) Number of weight Beta-diversity estimates of microbiota. (B) The results of ANOSIM analysis based on Bray-Curtis distance. [file Image_1.TIF]

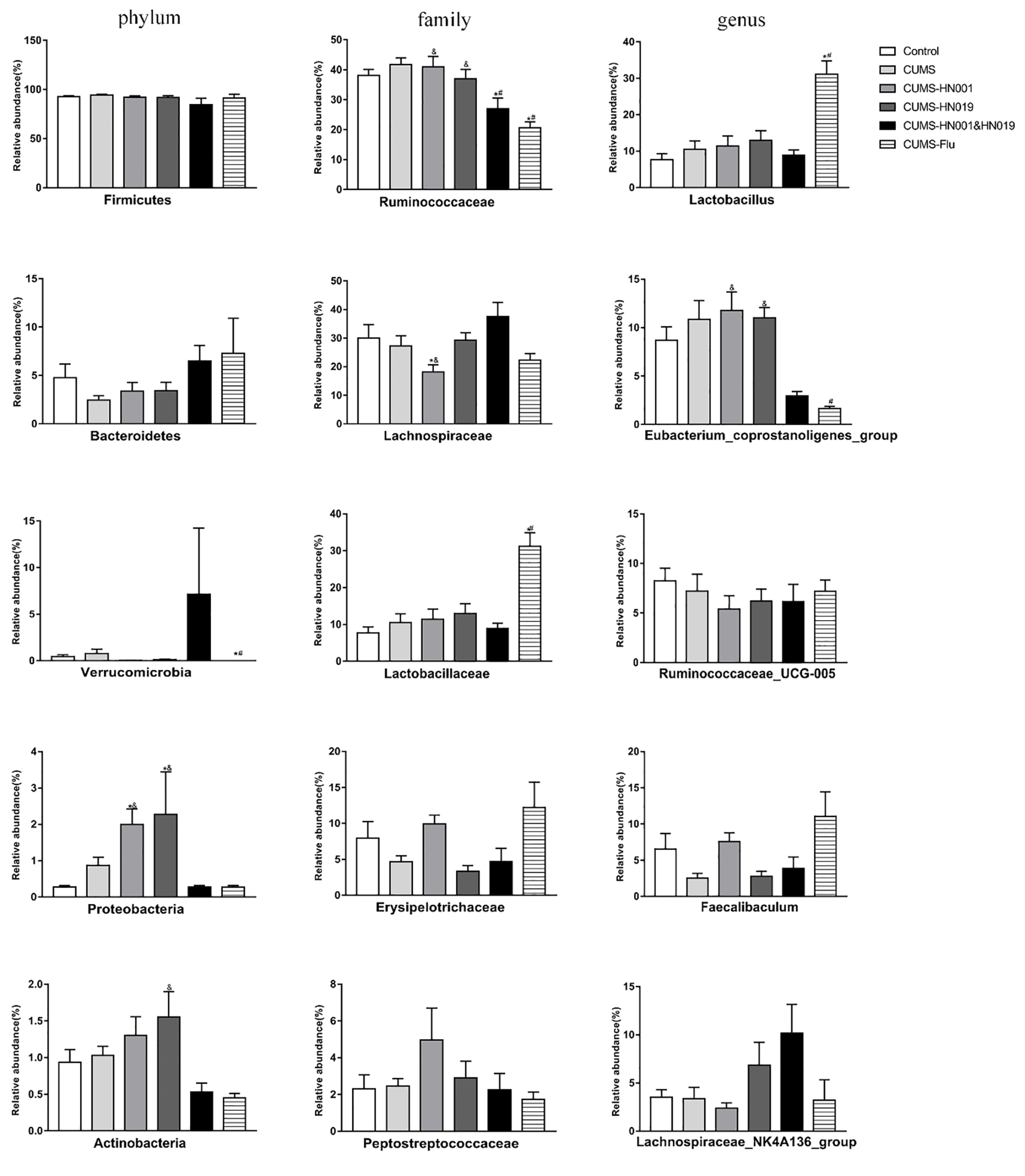

Supplement: Supplementary Figure 3 — Relative abundances of selected microbes at the genus level. Data are expressed as Mean ± SEM (n = 7). *p < 0.05 compared with control group. #p < 0.05 compared with CUMS group, &p < 0.05 compared with CUMS-HN001&HN019 group. [file Image_2.TIF]
